# H-Index Is Important for Postural Control for People with Impaired Foot Sole Sensation

**DOI:** 10.1371/journal.pone.0121847

**Published:** 2015-03-25

**Authors:** Shuqi Zhang, Brad Manor, Li Li

**Affiliations:** 1 Louisiana State University, Baton Rouge, LA, United States of America; 2 Georgia Southern University, Statesboro, GA, United States of America; 3 Division of Gerontology, Beth Israel Deaconess Medical Center, Boston, MA, United States of America; 4 Harvard Medical School, Boston, MA, United States of America; 5 Institute for Aging Research, Hebrew SeniorLife, Roslindale, MA, United States of America; 6 Key Laboratory of Exercise and Health Sciences, Ministry of Education, Shanghai University of Sport, Shanghai, China; Purdue University, UNITED STATES

## Abstract

**Introduction:**

People with Peripheral Neuropathy (PN), especially those with impaired sensory inputs through the small-afferent fiber (type II afferent fibers) reflex loop (SAF), might depend more on the large-afferent fiber (type I afferent fibers) reflex loop (LAF) for postural control.

**Purpose:**

To examine whether the function of the LAF reflex loop, reflected by the H-reflex and ankle joint proprioception, influences postural control when the SAF reflex loop is impaired, as indicated by reduced foot sole cutaneous sensation.

**Methods:**

Thirteen participants (8 women, 5 men) diagnosed with PN and 12 age-matched controls (7 women, 5 men) completed the testing protocol. Measures of interest included the H-index, active (AAP) and passive (PAP) ankle proprioception, plantar pressure sensitivity (PPS), average sway velocity (V_AVG_) and area (A_95_) during 30 seconds eyes-closed standing, 6-minute walk distance (6MWD) and timed up-and-go duration (TUG).

**Results:**

Statistically significant group-dependent regression was observed between V_AVG_ and H-index. Compared to the control group, the PN group demonstrated reduced PPS (2.0 ± 1.9 vs. 4.2 ± 1.2, *P* < .05) and H-index (63.6 ± 10.9 vs. 76.4 ± 16.0, *P* < .05), greater V_AVG_ (3.5 ± 2.1 vs. 1.6 ± 0.6cm/s, *P* < .05) and A_95_ (10.0 ± 10.1 vs. 2.5 ± 1.5cm^2^, *P* < .05), shorter 6MWD (442.2 ± 93.0 vs. 525.3 ± 68.2m, *P* < .05), and longer TUG (9.4 ± 1.6 vs. 6.5 ± 1.3s, *P* < .05). Within the PN group, but not the control group, the H-index was correlated with V_AVG_ (r = -.56, *P* < .05). Moreover, within the PN group only, PAP scores were correlated with 6MWD (r = -.68, *P* < .05) and TUG (r = -.59, *P* < .05) performance. No other statistically significant group difference, correlation or group-dependent regression was observed.

**Conclusion:**

V_AVG_, 6MWD, and TUG correlated with LAF reflex loop function observed among those with impaired functioning of the SAF reflex loop. This observation suggests that the LAF reflex loop may be critical to the control of balance in those individuals suffering from small-fiber PN.

## Introduction

The prevalence of all-cause peripheral neuropathy (PN) is 2.4% in the adult population and 8–10% in those over the age of 55 [1]. PN is a neurodegenerative disease that damages the peripheral nervous system in a distal to proximal fashion [1]. People with PN often exhibit nerve conduction impairments and associated paresthesia (numbness, pain, and tingling sensation) within the lower extremities [2]. These PN-related neurological abnormalities are believed to disrupt postural control [3, 4] and diminish functional mobility [5], which are both important patient outcomes linked to survival [6, 7] and fall risk [8, 9] in older adults.

Postural control is a complex, dynamic process that involves neuromuscular coordination, biomechanical interactions and multiple sensory feedback loops. Somatosensation is critical for postural control [10]. Pressure-related feedback arising from cutaneous receptors in the foot soles contributes to the regulation of postural sway in both young and elderly individuals [11–16]. Ankle joint proprioceptive information is also believed to contribute to postural control [17–19]. While the acute effects of experimentally-reduced ankle joint proprioception on postural control have been inconsistent [20, 21], chronic deficits in ankle joint position sense are an independent risk factor for falling in older adults [4]. Additionally, the stretch reflex arc, which functionally connects afferent fibers within muscle spindles to α-motoneurons, contributes to lower-extremity motor responses during both standing and walking [22, 23].

In the presence of chronic somatosensory impairments, postural control appears to depend more upon other, intact sources of sensory feedback [24–26]. For example, individuals with PN are more depend upon vestibular feedback, as their postural control is more sensitive to vestibular perturbations as compared to age-matched controls [27]. Tactile and proprioceptive information from the foot soles and ankle joint flexor muscles are believed to be co-processed following a vector addition mode to sub-serve the maintenance of erect stance in a complementary fashion [28]. Furthermore, in healthy adults, experimental reduction of plantar cutaneous sensation leads to increased muscle activity about the ankle joint when standing, thereby suggesting that ankle joint and muscle proprioception may serve a compensatory role in this situation [29].

Cutaneous receptors and secondary muscle spindles, which are primarily responsible for foot sole sensation and perception of muscle length, appear to be more important for postural control than primary muscle spindles and the Golgi tendon organs, which are primarily responsible for perception of vibration, muscle velocity, and joint position [30–35]. As sensory receptors are stimulated, nerve impulses arise and propagate into the spinal cord along reflex arcs that induce corresponding muscle contraction. The type Ia afferent pathway innervates primary afferents of the muscle spindle, whereas the type II afferent pathway innervates secondary afferents in muscle spindles and mechanoreceptors under the skin [22, 23]. These two corresponding reflex loops have often been examined by stretching the soleus muscle to obtain two bursts of muscle activity. The moderate latency component of the stretch reflex (MLR), which has an onset latency of approximately 70ms and is mediated by type II afferent fibers [36], contributes more to postural and locomotor control as compared to the short latency component of the stretch reflex (SLR), which has an onset of approximately 40ms and is mediated by type I afferent fibers [37–39].

PN does not selectively affect plantar cutaneous receptors alone but rather impairs all peripheral sensory systems and is accompanied by significant reductions in nerve conduction velocity [2, 40]. Diminished foot sole pressure sensation related to impairment to the SAF reflex loop is the most common symptom of PN and believed to be the primary threat to postural control [31, 32, 41]. The contribution of the LAF reflex loop to postural control, particularly in those with poor cutaneous foot sole sensation (i.e., SAF reflex loop impairment), however, is unknown.

In this study, we investigated the contribution of the LAF reflex loop, as assessed by the Hoffmann reflex and ankle joint proprioception, to postural control in people with and without impaired SAF reflex loop function, as indicated by diminished plantar cutaneous pressure sensation. The Hoffmann reflex test estimates the function of the stretch reflex arc [42, 43] and is minimally affected by peripheral sensory inputs. We further investigated the correlation between the integrity of the SAF and LAF reflex loops and functional mobility. We hypothesized that 1) the strength of correlations between sensory variables and the measures of postural control may be significant in PN group but not in the control group; 2) the conduction time of H-reflex and ankle joint proprioception may moderate the relationships between group and measures of postural control in standing and walking.

## Methods

The original study recruited 23 people (16 women, 7 men) diagnosed with PN and twelve age-matched healthy people (7 women, 5 men). For the secondary analysis, participants who were unable to finish the 6MW test (2 people), absence of H-reflex (5 people), and with intact plantar pressure sensation measured by the monofilament (3 people) were excluded. Participants provided written informed before tests. This project was approved by the Louisiana State University Institutional Review Board.

### Procedures

Participants’ age, height, body mass, cause and duration of diagnosed PN were recorded. A testing battery was completed, which included assessments of plantar pressure sensitivity, H-reflex, active and passive ankle proprioception, standing postural control, functional capacity and mobility. At least 3 minutes rest was provided after each test.

### Plantar Pressure Sensitivity (PPS)

PPS was assessed using a 5.07 gauge Semmes-Weinstein monofilament (North Coast Medical. Inc, Morgan Hill, CA, USA) with the participant lay supine on an examination table. Testing sites included the heel (HL), mid-foot (MF), along with the bases of first (M1) & fifth (M5) metatarsal, and big toe (BT). Specific protocol details can be found elsewhere [44].

### H-index test

H-reflex, as a reliable neurological measure for people with PN, was completed using two, 20 mm diameter recording electrodes (EL503, Vinyl 1-13/8”, BIOPAC Systems, Inc. Goleta, CA, USA) placed in parallel to the orientation of muscle fibers at the belly of the right lateral gastrocnemius. Inter-electrode center distance was 20 mm [45]. The test was performed with the participants lying prone with their feet hanging slightly off of the edge of the examination table. The reference electrode was placed on the Achilles tendon on the same limb. The electrode fixation sites were cleaned with alcohol gauze to reduce impedance. Once set-up was completed, the tibial nerve of the right popliteal fossa was stimulated by a 1 ms square-wave targeting the tibial nerve using a bipolar constant voltage stimulator (BSLSTMA with the MP30, BIOPAC Inc., Goleta, CA, USA). Resulting electromyographic (EMG) signals were amplified and recorded by a MP36R Data acquisition and analysis systems (BIOPAC Systems, Inc., Goleta, CA, USA). Stimulus intensity was increased progressively from zero volts in 5 volts increments until concurrent H- and M-waves were observed. The latency between each H- and M-wave was recorded nine times. The average of these nine latencies was used to calculate H-index, where, H-index = ${\left[\frac{Height\;\left(cm\right)}{\Delta t_{H}-\Delta t_{M}}\right]}^{2}*2$ [46].

### Ankle Proprioception

Active (AAP) and passive (PAP) ankle proprioception tests were conducted using a Biodex 3 dynamometer and the Biodex Advantage Software Package (Biodex Medical System, Inc., Shirley, NY, USA) following a protocol deemed reliable for older adults with PN [45]. Participants sat in a Biodex chair reclined at 70° with legs parallel to the ground. The right ankle joint was properly aligned with the axis of the dynamometer, and the right foot was fastened securely by a Velcro strap to the ankle Inversion/Eversion attachment. The weight of the limb was supported by an additional attachment placed under the thigh. The testing procedures of active and passive ankle joint repositioning consisted of localizing three target positions: 15° of inversion, 0° subtalar neutral, 10° of eversion [47]. Prior to testing, participants had opportunity to practice. During AAP test, participants started from maximal inversion and then move their ankle joint to the targeted position. The order of target position was randomized, and each position was tested three times, following a position specific practice session.

Passive ankle reposition was examined in the same manner except that the attachment moved the foot at 2°/s from maximal inversion to the target position during the practice session. The dynamometer was stopped at each target position for 10 s, during which time each participant was instructed to concentrate on the position of the ankle joint. During the test session, the ankle was passively rotated in a similar manner as the practice session. Participants were asked to stop the motion of the machine when they believed their ankle reached the target position by pressing a hand-held trigger button. In order to avoid possible timing-prediction learning effects, the specific velocity of ankle joint movement was not described to the participant. AAP and PAP was determined by the average absolute error, in degrees, between the preselected angle and the repositioned angle [45].

### Standing Postural Control

Standing postural control was assessed with a force platform (AccuSway, AMTI, Watertown, MA, USA). Participants stood on the force platform facing forward with their heels about 10 cm apart and were instructed to stand as still as possible. Center of pressure (COP) position data were sampled at 50 Hz during one 30s trial with eyes closed. Average of sway velocity (V_AVG_) and 95% sway area (A_95_) were calculated [48].

### Functional mobility tests

The 6-minute walk distance (6MWD, in meters) and timed up and go (TUG, time in seconds) tests were used to represent functional mobility [48]. In the 6-minute walk test, two cones were set 30 meters apart in the hallway. Participants were required to walk between in then around each cone as fast as possible for six minutes. They could use the walking assistance if needed. In the TUG test, participants were seated against the back of the arm chair with a cone 3 m in front of the chair. On the word “go”, participants were asked to stand up using the arm rests if needed, walk around the cone, and sit back against the back of the chair. The timer was started when participant’s back left the back of the chair and stopped when the participant’s back first touched the back of the chair. The time to complete the 3 trials was recorded and averaged.

### Statistical analysis

Statistical analyses were performed using SAS 9.3 (SAS Institute, Cary, NC). Descriptive statistics (mean ± SD) were used to summarize all numeric variables. Potential group differences in demographics were examined by independent t-tests. Potential group difference in PPS, H-index, A_95_, V_AVG_, 6MWD, TUG, PAP, and AAP were examined using one-way MANOVA. Univariate ANOVA was followed where necessary. To test the first hypothesis where group differential correlation exist between measures of sensory and postural control in standing and walking, we used Pearson correlation analyses

To examine the second hypothesis that the sensory variables may moderate the relationship between group and the postural control in standing and walking, we used the homogeneity of regression slopes test. We adopted general liner regression to compare the regression lines (slopes and intercepts) of dependent variables (mobility/postural control) and independent variables (H-index/proprioception) between two groups with the following model: V_AVG_ = b_0_+b_1_*group + b_2_*H-index + b_3_ *(group* H-index). Different variables were examined by the same model. The slopes difference was examined by the significance of the interaction term between group and independent variables (i.e., H-index/proprioception). If the interaction terms were statistically significant, it indicates the regression lines are not parallel with each other, which means the strength of the statistical relationship between the two variables is dependent upon the third variable (group). Therefore, the statistically significant interaction term would be used to test the second hypothesis [49, 50]. An alpha value was set at .05.

## Results

Of the 23 PN participants recruited for the study, data from 10 subjects were excluded from the present analysis due to inability to finish the six-minute walk test (n = 2), inability to induce the H-reflex (n = 5) and lack of plantar pressure sensitivity impairment (n = 3). Other than the history of PN, demographic characteristics were similar between the two groups (See Table 1 for more details).

**Table 1:** Demographics and Outcome Variables for PN and Control Groups.

	PN (N = 13)			Control (N = 12)			*P*-value	Effect Size
	Mean		S.D.	Mean		S.D.		
**Age (years)**	73.0	±	8.0	70.5	±	9.5	.4146	
**Height (cm)**	165.3	±	10.3	167.7	±	8.8	.5318	
**Body Mass (kg)**	79.4	±	20.6	72.0	±	14.4	.3113	
**PPS**	2.0	±	1.9	4.3	±	1.2	.0018*	.35
**H-index (cm** ^**2**^ **/ms** ^**2**^ **)**	63.6	±	10.9	76.4	±	16.0	.0272*	.20
**PAP**	6.5	±	3.5	3.9	±	1.4	.027	.20
**AAP**	7.7	±	5.3	5.6	±	2.8	.235	
**6MWD (m)**	442.2	±	93.0	525.3	±	68.2	.0187*	.22
**TUG (sec)**	9.4	±	1.6	6.5	±	1.3	<.0001*	.46
**V**_**AVG**_ **(cm/sec)**	3.5	±	2.1	1.6	±	0.6	.0060*	.30
**A**_**95**_ **(cm** ^**2**^ **)**	10.0	±	10.1	2.5	±	1.5	.0203*	.30

*Indicates statistically significant difference between the two groups at level of .05.

The PN group demonstrated reduced PPS, greater repositioning error in PAP test and lower H-index than the control group. The PN group also exhibited greater V_AVG_ and A_95_, shorter 6MWD and prolonged TUG compared to the control group.

### H-index

**Fig 1:**
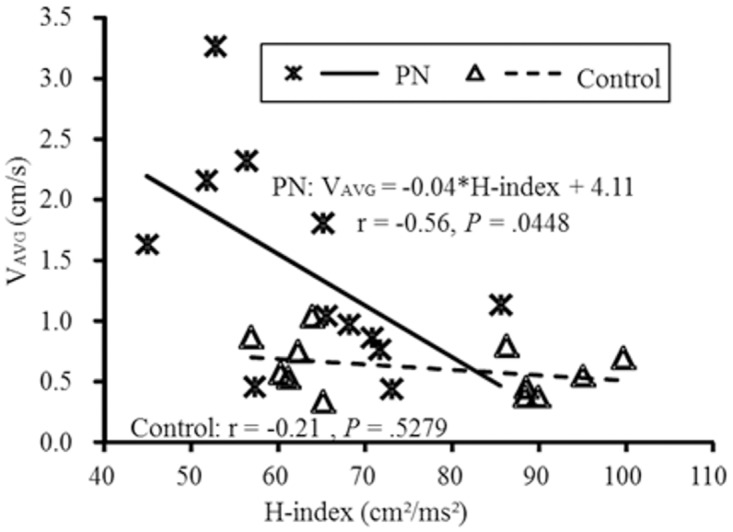
The regression lines between average sway velocity (V_AVG_) and H-index with raw data for the two groups are displayed. The relationship between V_AVG_ and the H-index was dependent upon group.. The least square equation of regression line for PN group is listed on the top, which indicate greater H-index was associated with slow sway within the PN group but the not the Control group. Correlation coefficients and P values for both groups are listed in the figure.

A significant correlation was observed between V_AVG_ and H-index in the PN group (r = -.56, *P <*. *05*), but not in the control group (r = -.21, *P =* .53; See Fig 1 for more details). Individuals with a shorter conduction time of H-reflex exhibited less sway velocity in the PN group. No significant correlation was observed between H-index and other physical performance outcomes in each group. Moreover, a significant interaction term of V_AVG_ was observed between H-index and group (F_1, 23_ = 9.59, *P <*. *05*). The conduction time of H-reflex moderated the relationship between group and V_AVG_, which indicates PN individuals with a shorter conduction time of H-reflex exhibit less V_AVG_ compared to the controls. Although the mean V_AVG_ of the PN group was greater than the control group, this difference diminished with an increased H-index (See Fig 1 for more details). No other statistically significant interaction terms were observed between H-index and group in any of the other measures of physical performance. Therefore, H-index was not only correlated with sway velocity in PN group, but also moderated the relationship between group and V_AVG_ during quiet standing.

### PAP

**Fig 2:**
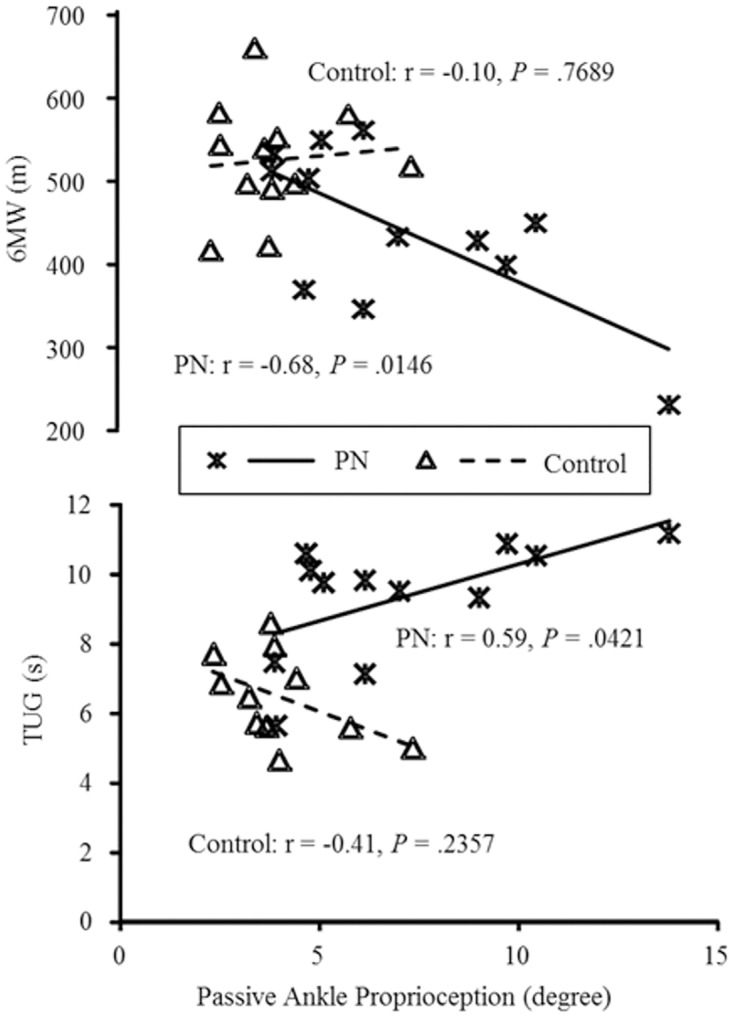
Passive ankle proprioception (PAP) in relation to 6-minute walking distance (6MWD, top panel) and Timed up and go (TUG, lower panel) are displayed here. In the PN group, but not in the control group, those with less repositioning error tended to perform better (longer distance in 6MWD and shorter time in TUG) in each functional mobility test.

Significant correlations were observed between PAP and the 6MWD (r = -.68, *P <* .05) as well as the TUG (r = .59, *P <*. *05*) in the PN group, but not in the control group (PAP / 6MWD: r = -.01, *P* = .77; PAP/TUG: r = -.41, *P* = .24; See Fig 2 for more details). Specifically, within the PN group only, those with better passive ankle joint proprioception demonstrated better performance in the 6MWD and TUG tests. No significant correlation was observed between PAP and the other outcomes of physical performance in either group. No significant interactions were observed between PAP and group on the outcomes of physical performance.

### AAP

No significant correlation was observed between AAP and measures of physical performance in either group. No significant interactions were observed between AAP and group on measures of physical performance.

## Discussion

The PN group exhibited exaggerated body sway, greater sway velocity and reduced mobility compared to the control group, which is consistent with previous studies [5, 48]. A shorter H-index was expected for PN group, since reduced sensory nerve conduction velocity is typical for this population and contributes considerably to the H-reflex arc. The PN group demonstrated greater reposition errors in the PAP test, but similar reposition errors in the APP test, compared to the control group [51, 52].

Results of the current study suggest the H-index was the moderator of the relationship between average sway velocity and group. It suggests that the group difference of average sway velocity depends on H-index. The H-reflex conduction time was the moderator for postural control among individuals with PN who suffered plantar pressure sensation impairment. The average sway velocity was independent from H-index evidenced by the negative results of correlation test. The moderating effect suggests that PN individuals with a greater H-index may exhibit similar postural control capacity as healthy controls. Therefore, the conduction time of H-reflex moderates postural control for individuals with PN who have suffered plantar pressure sensation impairment.

H-index is the normalized time course between the onsets of the M- and H-wave relative to an individual’s height. It represents the latency of the LAF reflexive loop, including the transmission time of the large-diameter peripheral nerve (sensory and motor) and the synapses time in the spinal cord [53]. One explanation why the H-index moderated posture is that the relatively intact function of H-reflex would compensate for the sensory impairments in the feedback control loop during quiet standing. People with PN tend to rely more on the alternatively available sensory resources at both the peripheral and central levels of the nervous system. For example, the inaccurate detection threshold of ankle position while seated could be compensated by additional audio cues during the repositioning tests in PN [52]. At the central level, Manor’s group examined the correlation of brain volume and walking outcomes for diabetic peripheral neuropathy (DPN) individuals and healthy controls, where certain brain matter volume were only correlated with gait parameters in DPN group. The observed group-specific strength of correlation suggest that up-weighted suppraspinal elements of motor control to regulate walking outcomes among individuals with DPN [54]. As the standard and reliable measure of LAF latency [45, 46, 53], H-index represents the up-weighting component of the feedback control loop during the quiet standing in PN with reduced PPS.

To date, there is no documented correlation between sensory nerve conduction velocity and postural control outcomes. Nevertheless, one study suggested that the conduction velocity of the sural nerve could mediate postural control while quiet standing in people with PN [31]. Although the importance of SAF was highlighted in Nardone’s studies [32], the moderating effect of the H-index highlights another possibility that standing postural control depends on the coupling between the SAF and LAF reflexive loops. Therefore, the function of the LAF and SAF reflexive loops are acting as a compensatory mechanism to postural control during standing.

Significant correlations were observed between PAP and functional mobility in the PN group, but not in the control group. However, there was no significant moderating effect observed. This may be due to the small sample size. Slower walking speed is a compensation for postural control deficits among people with PN. Functional mobility does not correlate with strength but with postural control capabilities in PN patients [5] suggesting that the decreased walking speed was primarily due to sensory impairments. More accurate PAP indicates better functional mobility in PN [55–57]. Previous studies have shown that the proprioception from a single muscle, tendon, and ligament of the ankle joint dose not influence postural control during standing and walking [58–60]. The broader assessment of ankle proprioception demonstrates that comprehensive function of the ankle joint, including ankle proprioception and stability is an important indicator of comprehensive joint function for postural control [61]. The association between PAP and functional mobility in people with PN suggests that ankle proprioception is an active sensation responsible for postural control during walking.

APP was not statistically correlated with functional mobility in either group. The key difference between the active reposition and passive reposition tests may be a reliance on the different sensory information needed to spatially calibrate the final position [62]. Unlike self-induced movement, passively moving to the final position may depend more on information from muscle spindle, such as detecting changes in muscle length and velocity. In passive movements, people with PN have limited and inaccurate information from muscle spindles because of an impaired somatosensory system; whereas the control group would receive relatively normal information from muscle spindles. In active mode, people with PN can freely move their feet to generate sufficient muscle spindles activity.

In this study, we did not examine the conduction velocity of the SAF reflexive loop. However, the impaired plantar pressure sensation indicates the dysfunction of sensory receptors in the SAF reflexive loop. Also, this study only compared PN individuals with reduced plantar pressure sensation to the control group, without the people with PN but have intact plantar pressure sensation. Also, only healthy participants for the control group were virtually guaranteed to have relatively intact SAF and sensory receptors, which is more appropriate for this study.

Please note that the neurological and functional outcome variables reported here had a greater range in the PN group due to the chronic nature of their impairments. For example, the standard deviation of V_AVG_ (Table 1) was approximately 36% of the mean for the control group but nearly 60% for the PN group. The homogeneity of the control group may contribute to the observed differences in the correlations between groups, as observed in Fig 1.

## Conclusion

Overall, these observations provide the information that chronic effects of reduced PPS lead to adaptive changes in postural control in standing. People with PN depend more on the LAF reflexive loop and ankle proprioception for postural control when the SAF reflexive loop or its sensory receptors are impaired. Similarly, ankle joint proprioception and joint stability are more important to functional mobility in people with PN compared to healthy controls. This study suggests that interventions targeted at improving LAF and ankle proprioception could be effective treatments for people with PN.
